# African Immigrant’s Women Experiences on Extended Family Relations

**DOI:** 10.3390/ijerph19148487

**Published:** 2022-07-12

**Authors:** Philomina Okeke-Ihejirika, Neelam Saleem Punjani, Bukola Salami

**Affiliations:** Faculty of Nursing, University of Alberta, Edmonton, AB T6G 2R3, Canada; pokeke@ualberta.ca

**Keywords:** Africa, immigrants, refugees, participatory action research, extended family relations, postcolonial feminism, transnationalism

## Abstract

African immigrants are increasingly migrating to high-income countries, including Canada, in search of a better life. These immigrants often face several challenges, such as keeping healthy ties with their extended families back home and in a new socio-cultural context. We present findings from a participatory action research (PAR) study of Sub-Saharan African immigrants and refugees (SSAIRs) living in Alberta, Canada. Using the theoretical framework of postcolonial feminism and transnationalism, in this study we investigated how cultural roots and transnational ties affect newcomer transition and integration to provide information on the female perspectives of SSAIRs. The results of the study indicate that maintaining relational ties with one’s extended family in the homeland has been highlighted as both a source of support—providing moral, social, religious, and cultural support during the integration processes—and strain, with participants noting its impact on their integration processes, such as delaying the ability to restructure life and to successfully plan their life financially. Our findings outline various implications of the existing gaps and recommendations for policymakers and community stakeholders for future improvement. Overall, our study findings affirm the importance of extended family relations for African immigrants living in Canada.

## 1. Introduction

Sub-Saharan African immigrants and refugees (SSAIRs), often referred to as Black Africans, come from about 40 ex-colonies of mainly Britain (e.g., Kenya, Nigeria and Uganda) and France (e.g., Mauritania, Senegal and Côte d’Ivoire) and form the majority of African newcomers [1]. SSAIRs have attracted a relatively low profile in Canada, in part, because they are usually lumped with either the broader immigrant population or the equally growing pool of Blacks, which, at 1,198,540 in 2016, made up 3.5% of Canada’s population with 71% (aged 25 to 59) being foreign-born [2]. Interestingly, the rise in Canada’s Black population is largely fueled by SSAIRs in newer destinations such as the Prairies. In Alberta, for instance, the number of Blacks has grown significantly from 39,955 in 1996 to 174,655 in 2016 [3]. The proportion of SSAIRs grew from just 1.9% of newcomers to Canada before 1971 to 13% in 2016 [3], with Africa placing second among Canada’s immigrant-sending regions. This study, emerging from three years of research on SSAIRs in Alberta, Canada, contributes to addressing the gap in the literature on SSAIRs’ extended family experiences. The purpose of this study was to explore the experiences and viewpoints related to the extended family relations and family life of African immigrant women living in Canada.

Although they are socially diverse, SSAIRs share an affinity for community life rooted in religion and culture, both of which endow a deep sense of belonging and affirm collective responsibility [4]. Gender relations within the immediate and extended family form a bedrock on which the polity structures and executes collective responsibilities [5]. SSAIRs’ history and immigration trajectories differ notably from those of other Canadian Blacks and barriers to rebuilding their lives raise serious concerns. Unlike previous Black immigrant cohorts, such as those from the Caribbean, SSAIRs do not exhibit the high rates of single-parent, female-headed households, or of economically marginalized men [6], stemming from longstanding systemic inequities rooted in slavery and colonization. Similarly to the broader population of more recent immigrants, however, SSAIRS not only maintain their ties to countries of origin but also find ways to nurture these ties as a crucial facet of support, in a new homeland where they have arrived with little or no culturally appropriate support system. The extant literature on the impact of transnational ties on newcomer transition and integration in Canada is relatively small but steadily growing [7]. The little we know suggests that a critical understanding of these ties is crucial to the development of public policy and practice and meaningful research agendas to better serve immigrants [8]. SSAIRs, one of the newest and fastest growing Canadian immigrant groups, remain highly under-researched.

The concept of family connectedness is a prominent cultural feature in African Black families [7,8]. Immigration changes not only the family structure but also the relationships between household members. In the context of immigration, the importance of extended families is reflected in clustered patterns of migration settlement. Immigrant people often live in the same neighbourhoods or even the same apartment buildings in order to provide family support such as sharing meals and childcare responsibilities [9]. Married couples often share both paid work and housework in a more equal way than they would in their country of origin. African immigrants extend their lives back across the Atlantic, remaining in constant contact with their families on the continent. Most are very honest about longing for home and those they left behind. They usually keep in touch with their home countries in many ways [10,11]. For many immigrants, the telephone is the preferred channel of communication with home. Most immigrants call home frequently and many even reach their distant relatives back home. According to the participants, Immigrants also stay in contact with their families at home by sending “remittances”, or sums of money that they wire abroad using several different services. Overall, family support through the exchange of remittances is essential to African immigrants, particularly among working-class families [12].

The role of gender is prominent in regard to the immigration experiences of African immigrants [13]. In general, among SSAIRs, gender roles are traditional and patriarchal, with men and women assigned to separate scopes of work in which males are breadwinners and females are homemakers. This idealized gender role pattern prevails despite high rates of women participating in the labor force. Studies have noted that women’s employment post-migration tends to change gender role patterns, causing a relatively more unequal distribution of domestic tasks within immigrant families [13]. For African men, domestic activities are often seen as being unsuited with the notion of masculinity. The lack of support from a male partner within the household leads to extra and burdensome workloads for women and increased dependence on female relatives and female-based support networks.

In this study, female study participants identified specific ways in which ties to their families “back home” undermined their wellbeing within the nuclear family in Canada but emphasized, for the most part, the linkages they had established as mostly being key enhancers in the process of rebuilding life in a new homeland. Gender relations, the experiences and viewpoints of participants show, not only shape the dynamics of family life in Canada but also interact with transnational relations in different ways for men and for women.

### Theoretical Framework: Postcolonial Feminism and Transnationalism

We adopt both a historical and an intersectional approach in analyzing the experiences and viewpoints of our participants, drawing primarily on two feminist theoretical perspectives: postcolonial feminism and transnationalism. A postcolonial perspective rejects the homogenization of marginalized populations within and outside the West, a strategy that often subsumes their diverse histories and circumstances [14]. Thus, we recognize the diversity of histories, cultures, social contexts and circumstances of migration and settlement that define African immigrants. We focus, however, on the commonalities, which uniquely shape their personal goals, as well as their settlement and integration trajectories. These commonalities include (1) collectivist cultures that place community above the individual, valuing family, immediate and extended, as a crucial engine for enacting collective responsibility [15]. (2) Gendered and racialized by culture, colonization and capitalist expansion prior to their migration, albeit in different ways, women’s status remains socially subordinate to men’s in the transition into a postcolonial era [16]. (3) Due to tradition, women’s latitude to pursue economic ventures is curtailed, given their primary responsibility to provide for their children. The postcolonial perspective also highlights women’s cultural knowledge, agency, and resilience in engaging life’s challenges as crucial actors within and outside the immediate and extended families [17]. Although men’s social status as decision-makers and critical agents is similar to the case in most societies, the role men could play as allies with women in the process of establishing a home in a host society is crucial [18].

Transnational approaches to migration recognize two key trends that shape the lives of immigrants, particularly women. First, the flow of people and goods across national boundaries, propelled by forces of global capitalism and the exigencies of political upheaval, has compelled the reappraisal of life in Western host societies as geophysically stable and mediated only by forces within them [19]. Immigrants not only hold on to but seek ways to nurture ties to their homelands as they anchor themselves in new soil. Transnationality highlights aspects of an immigrant’s life that extend beyond the host society’s geophysical space and consider how they intersect with other social trends and categories [13]. Transnationals interact with cultural identities and practices in homelands, diasporic communities and their host societies. Transnational feminist approaches challenge a longstanding presumption that men and women are equally involved in making decisions about when and where to migrate, which has only been recently questioned [20]. Ties to countries of origin remain a crucial source of support, the sparse extant literature suggests, but for male and female spouses, relationships with extended family members could present unequal challenges [21]. These challenges could also be mediated by the highly gendered nature of international migration and settlement; decisions about the destination, timing and logistics of migration are highly gendered, often resting with male partners. Shifts in gender roles within the family can also easily upset gender relations, placing women in vulnerable situations [22]. However, these patterns are not uniform across cultures and contexts; thus, there is a need for further research that links structures of gender evolving from specific histories and changing social contexts to specific immigrant contexts.

## 2. Methodology

This study was conducted between 2017 and 2020. Our data collection approach relied on the participatory action research (PAR) framework. PAR engages researchers and participants as co-constructors of knowledge who work together to examine an issue to change it for the better [23]. PAR differs from most other approaches to public health research because it is based on reflection, data collection and action that aims to improve health and reduce health inequities through involving the people who, in turn, take actions to improve their own health [24]. The reflective process is directly linked to action, influenced by an understanding of history, culture and local context and embedded in social relationships. The process of PAR is empowering and leads to people having increased control over their lives [25]. A purposeful sampling strategy was used to recruit participants through self-administered online surveys developed by a research team, and assistance was provided by community workers and an established network of community organizations. The rationale for the use of purposive sampling is to select a group of individuals that are especially knowledgeable about or experienced with a phenomenon of interest. Particular efforts were made to include women and men; people from different religions, such as Christians and Muslims; and immigrants from various parts of Sub-Saharan Africa. We conducted individual interviews with 20 women and 20 men who identified as Sub-Saharan African immigrants living in Edmonton or Calgary at the time of the study. This paper only focuses on the experiences of women participants. In an interview, participants were asked to share their experiences about extended family relationships and the challenges they faced as an African immigrant. The three main questions asked during the interview were (1) Tell me briefly about your experience with extended family own your own side (Probes: view/nature of extended family, responsibilities, influence on nuclear family as support/stressor). (2) Tell me briefly about your experience with your spouse’s extended family (Probes: view/nature of extended family, responsibilities, influence on nuclear family as support/stressor). (3) Based on how you have fared with extended family, comment on how African families should manage their extended families (Probes: own/spouse’s family, comments for families in general).

The inclusion criteria included participants who were Canadian citizens or permanent residents, spoke English and were at least 25 years of age. We excluded people who were international students, temporary foreign workers or refugee claimants due to unique policies that influence their circumstances. We conducted face-to-face or phone semi-structured interviews as per participants’ preferences. Informed consent was obtained from the participants and all the interviews were audio-recorded and transcribed. Confidentiality and privacy were maintained by using encrypted computer-based files, and documents (i.e., signed consent forms) were stored in a locked file cabinet and all personal identifiers were removed from study documents. Interview transcripts were thematically analyzed using NVivo software [26]. Rigor and trustworthiness were maintained through ensuring credibility, transferability, dependability and conformability in the research process [27]. Ethics approval for this study was obtained by the University of Alberta Research Ethics Board.

## 3. Results

The following themes emerged from the manual coding and analysis of the study transcripts: (1) Extended family as a source of crucial support, a sense of belonging and collective responsibility; (2) managing extended family as a challenge in a new homeland; and (3) the burden as well as the privilege of extended family according to men. In addressing this challenge, participants highlighted (4) improved gender relations through open communication and the need to set boundaries.

### 3.1. Extended Family as a Source of Crucial Support, A Sense of Belonging and Collective Responsibility

Participants spoke about the importance of support systems and networks, particularly related to maintaining contact with family members and establishing communities with regards to parenting. The most important issue for immigrant Africans was balancing nuclear family obligations with extended family obligations and navigating the tensions that this can create within immigrant families. In this regard as well, participants noted the importance of clear communication between partners to ease potential tensions from the perceived sense of obligation in the African culture to maintaining extended family support networks and providing financial support to those networks. One noted the team effort between her and her spouse:

“Yeah my husband’s side like for me, for us both families, like we don’t divide, me and my husband we work together, like he looks at my family as his own, and even his family there are very nice to me and they are my family; but unfortunately for my husband he lost all his siblings, he’s the only one surviving, so the siblings left their children that’s—and he’s the firstborn of the family … so there is an obligation, yes”.

(CAF013)

Participants also spoke on the sense of obligation in general to extended family networks and support systems:

“The extended family members always want that you should take care of—Like I said, at home I have so many people in my home. Some of them were extended families … What they expect is they expect you to help them in either learn a trade or put them in school. So I’ve been experiencing that. It’s still my passion of helping out, so at times I help out in my family and my extended family. That burden is always there. So you help out with your family and help out with the extended family all the time … I think the problem is we just grew up with this thing of we enable each other. We call it Ubuntu in Southern Africa …”

(CAF009)

“I think most of the Africans though I’m not the spokesperson for all African but like the way I see from even our people because we have a sense of family, right, we have a sense of family and we like tending to each other and we like holding each other up, we like pulling each other up … so you have to do what you need to do to help your people back there, you know, you have to share the little you have to share with them from time to time; so it is not necessarily sometimes every month, right, so you, of course, interested in maybe I need to help my people properly so you find that you may be a little bit, right, because of finances because you have your own responsibilities here also to take care of”.

(CAF012)

“… we as Africans as well, we have to look after everybody back home, you know. We are the breadwinners, we are overseas, everybody’s expecting too much from you. The funeral, everything is you. So I think yeah, most of the tension comes from that. Which makes us who we are. We are African, like we look after each other so, you know. In a way it’s bad but in anyways that’s who we are”.

(EDF011)

Study participants also expressed the importance of clearly communicating to family members the shift in gender roles (if/when it occurred), in which the woman had become the primary earner, contradicting the African notion of the man as the main provider for families. This was noted as a way to manage expectations from extended family members and highlighted some of the communication challenges that may exist within immigrant families and their support networks. A participant explained:

“Until Africans begin to know that they must have sincere transparent conversation around their finances, they will always have a problem. Because for example, if my husband brought me to Canada and his brothers heard he’s the rich one, but I could come to Canada and I’ll have a better job. It is my husband’s place to let his family know, oh I brought my wife to Canada, but she’s been lucky, she’s the one who has a job, do you understand, she’s the one. But how many Nigerian families are going to tell their families in Nigeria that their wife is feeding them, it’s a matter of communication, transparent communication, open communication, put it in the table and talk about it, and then there will be no problem”.

(EDF006)

Participants also noted that although African immigrant families in Canada tended to provide financial support to their family networks, they also benefited from the emotional and community networks maintained through interactions with their support systems in their countries of origin. Participants noted praying together, sharing experiences and seeking guidance/discussing issues. A participant put it this way: “in terms of relationship, we are still there, you call your sisters, you speak to them, you pray together and now it doesn’t change much, it’s just that you try to share the good experiences and the things you have learned here with them. And somehow, for example, like my own family, they listen and they understand …” (EDF006). Another participant, discussing the benefits, stated:

“Oh my gosh. We [extended family] have phenomenal, phenomenal communication. We [extended family] have WhatsApp groups and just my sisters, my mom and I and then other extended families, my aunts, because as I said, Zimbabwe is different from Canada. All my aunts are my little mothers. So even when I’m having a problem they’re the first person I talk to. So that’s—my cousins are my sisters and my brothers, so we have that relationship. A really good relationship”.

(CAF013)

The importance of community was also emphasized in regard to parenting and creating similar communal support systems, enjoyed in African countries for immigrant families in Canada as a way to ease and address the challenges of raising children in a different culture. Regarding parenting challenges and conflicts with children, a Sudanese participant explained the importance of a support system and community in avoiding such challenges:

“[O]ne of the issue that I see, that caused most of these things to be happening not right isn’t for the African child, it’s from the bigger people, it’s the parents. We don’t like ourselves, we don’t come together … Some of them because of the barrier, the language barrier, and then until—you cannot stop children from growing, they keep growing and the parents don’t have the language, you know, because like our people so many of them came because of the war and they became maybe directly from villages, they just ran … and they come here … they can’t be able to help their own child even with the homework, right, so the community should be able to, you know, they should be able to have groups where they have women that we can discuss not only women, even with men, how or what they want to see in the lives of their children because children they grow”.

(CAF012)

Aside from challenges based on expectations, participants expressed how Canadian ideals have influenced this relationship as well and allowed them to encourage family members to become self-sufficient and communicate the same message to each spouse’s network to avoid tensions within the family.

“But in terms of relationship, we are still there, you call your sisters, you speak to them, you pray together and now it doesn’t change much, it’s just that you try to share the good experiences and the things you have learned here with them. And somehow, for example, like my own family, they listen and they understand and sometimes they, you know, they give me credits, they applaud what I have learned here and then they try to use it and to, you know, they try to use it there, back in Nigeria. Try to apply it to their lives as well”.

(EDF006)

Some other participants noted similar experiences of encouraging family members to be self-sufficient and supporting them in that endeavour:

“… one of the things that I did was that I send some money for my sister to get some training, so she’s doing, like, a nurse aid role and she’s hoping that she’ll have a job now. That would mean that I can cut down on sending her money and start putting money aside for me for my retirement. Because my husband is already retired …”

(CAF013)

“… whatever we send from here, I think it’s supposed to be enough for them to start their life, so that they will not be dependent. They are supposed to do businesses, do something that will generate income for them. So, not to rely totally on those who are abroad”.

( EDF004)

### 3.2. Managing Extended Family as a Challenge in a New Homeland

In addition, participants touched on the challenges that come with interactions with extended family in countries of origin, particularly relating to the notion of improved economic status based on relocation to Canada and how this creates unrealistic expectations and pressure for support:

“So in terms of my relationship with my own extended family, I feel like we are pretty close, although we are in different countries. I’m obviously very far away. Most of them are in South Africa and Zimbabwe. The challenge that I would say I have had since moving here is most of them just think that now I’m in Canada I have a lot of money … So I’ve been getting a lot of messages, voice messages, phone calls, about people who are just asking for money for some reason or another. That’s the only challenge, that people now think I have a lot of money”.

(CAF007)

In addition, another also stated:

“The drawback is the constant demands, they cannot—they don’t get it really and I don’t blame them because back home they—we support each other. They were there when I was in school, I went to my sisters and they will always give you pocket—I expected that from them. If they didn’t give me I didn’t feel happy. I felt it was their duty to help me while I was in school, and they did that very well. Some of them didn’t eat, one of my sisters, her husband was even—she will ask me, “Go to this store, go and take all the provision you need for going back to school, I will give you money”; and she will do that from the little pocket money. She has some money from the food money her husband had given her and she could pay for that for two months. I looked after the time I could help her but unfortunately, I’m here and my finances can’t do even for my family not to talk of helping-. Right now I don’t even talk with her.; I don’t feel happy that I can’t help her”.

(EDF015)

Most participants agreed that while maintaining a connection with families in their countries of origin had the tendency to create undue tensions, managing communications and setting boundaries helped to manage any potential conflicts that may have arisen between partners in this regard. They also noted prioritizing their family/spousal needs before attending to maintaining ties with their extended family in the country of origin and supporting them. A participant commented that, “I think they [partners] should not feel responsible for every need and I think they first take care of themselves before they invest in taking care of other people because sometimes you can take care of other people and you realize that you neglected certain aspects of your life. You did not invest in yourself and sometimes it can affect even your health or your relationships so I think it’s good to first take care of themselves and do it in agreement together with the—like if they are married, the husband and wife, to be in agreement to do that then it will not cause you tension”.

(CAF014)

Participants discussed the various circumstances that contributed to the need to set boundaries so as to ease financial burdens within the home which had the potential to strain gender relations, stemming from providing support to extended family and community networks. Generally speaking, there was a perception of constant demand and dependence:

“What I find is that when we come to the diaspora we tend to over-help our families back home. And I find that this is not good. Because if it’s a case of giving them the fish instead of giving them the fish or teaching them to fish. And I’m only learning that now. You know, in the last year when I’ve been dealing with my sister and trying to get her to start becoming more independent and looking after herself and her family back home in Zimbabwe. But I think that’s what we really need to do is start becoming a lot more firmer with the people back home. Because they think that money grows on trees when—when they hear from us it’s all about money, or if they phone us it’s all about money …”

(CAF013)

“So really a lot of things we have had to do to support the extended family, helping my mom—I only have my mom—and then my siblings, paying school fees for their kids and everything. Sometimes from, you know, even providing money for food or things like that. So yeah, it has been cumbersome”.

(CAF014)

Participants also elaborated how the overdependence of extended family networks may sometimes hinder their development and growth, which further reinforced the need to set boundaries while maintaining those network systems. One participant noted that some of the support requests often seemed unreasonable based on the shared sense of obligation Africans have to support their community:

“I think what we do is a lot of Africans or a lot of African families, they expect too much from one another … Sometimes some of the requests are genuine like maybe someone is sick and stuff. But most of the requests, to be honest, are just quite unreasonable. And they hinder us Africans from reaching our own personal goals, then you end up also having resentment towards some of your family members”.

(CAF009)

Beyond establishing boundaries, participants noted the need to enforce the boundaries as a united front and communicate this clearly to extended family back in the country of origin, as well as to help manage expectations and relationships and ease the burden. One participant explained:

“Like everything is different but setting limits sometimes kind of helps. Sometimes you need set limits to say “You know what, maybe this year this is what I can give, what I can afford to give” but everybody has to look after themselves. “You can’t all depend on me all the time because I have my own things to do as well.” But setting limits I think, you have to set limits, you have to explain to them because most of them they think we are in Canada “Oh my goodness, money rains everywhere” but you just have to set limits. You say “This is this” or “This is who I’m helping.” That’s my thing, setting limits”.

(EDF011)

Participants also noted how boundaries were important in maintaining the support system and relating with extended family while managing family finances and gender relations:

“So in that regard, I always tell people you can’t do without your extended family back home, but you have to set the boundary. You need to let them understand that you are not making a million dollars here; if they are placing an unnecessary financial burden on you, you need to set the boundary and let them know, “I will do what I can within my means, but I can’t steal”, I’m sure they don’t want you to end up in jail. But if you don’t set those boundaries and be truthful about it, then people’s expectations of you are higher than what you can meet, and then they feel you are not helping them; and this is something you have to talk with your husband, you have to agree first as a family and you are both passing the same message to both families. So it’s not like you are doing so much for your husband’s family when your own family needs so much and you are neglecting them”.

(EDF006)

### 3.3. The Burden as Well as the Privileged of Extended Family for Men

Participants also spoke about the pressures their partners experienced to be providers for both nuclear and extended families. The general perception was that although men enjoyed some privileges based on the African culture as the head of the home, this position also carried considerable burdens. Given the changing dynamics of immigrant families and women taking more roles in sharing family responsibilities as providers, the perceived burden on men and their privilege was diminished to an extent. One participant commented on the burden and how her involvement upon migration to Canada eased some of the tensions:

And like I said, my husband carries the, most of the economic burden, financial burden and I just support with a few bills here and there, you know, but at least we now, we both know what we earn and then how to save when children’s school, when it’s time to send the children to school, we know what’s going to happen and we kind of know what we have.

“Yeah. When I got here, I was just really to work, like I worked with DynaLIFE, in their laboratory in the specimen department. I know that I, I used to work through the agencies, I worked in the bank, but they were very little money like I wasn’t even contributing, my husband was running the whole finances in the house, because he was a professor in the university, so. But I just used to support him very, in a very little way, unspoken support, like you know, he gives you money and then you add your own or whatever. So, that was how it was until I got my job until I went back to school; I took out a student loan anyway, so I went back to school. And then when I got here, I now took over some of the expenses, like some of the bills I had to pay, but generally, my husband pays most of the, carries most of the financial burden, so, in the house”.

(EDF006)

Similarly, other participants noted the perceived financial burden and pressures of responsibility African men felt to manage their nuclear and extended families;

“I think as a man, oh yeah absolutely. I think men, yes, because as a man your family they’re looking at you, you know, for support especially where families where the dad has passed on and you are the eldest son or you are the one who’s—we had the opportunity to leave, you know, Africa and go somewhere. So everybody’s like looking to you as a man. But yet you the woman too who’s saying, you know, “You have to look after me” and she has her own people to look after. But I find it more with men than women. Extended families, they expect more”.

(EDF011)

“[S]o it is my husband only who is working to support the family. So sometimes it is tough, you don’t have money, even if they request. He said, “that’s our relationship”. Sometimes they are not happy because for them, we are here, they ask us for money, we don’t have money, they spend like two/three months without calling him because they didn’t get the money they asked for. That is okay. We– we try to live with that. And when we have money, we just send it to them”.

(EDF003)

“… With my husband’s side, now I feel like they also want us to take responsibility of taking care of his brothers, meaning that they have to come here to Canada. Not sending money, this one now different. They want to send like his brothers also to come here so that we can stay with them. Which I think is not a bad thing, but we might just not be ready financially”.

(CAF007)

### 3.4. Improved Gender Relations through Communication

The study revealed that improved communications between spouses have been a large contributor to improved gender dynamics within the family. Participants spoke about the difference between the culture of communication between Africans and the level of transparency and conversation that was required while settling in a foreign country. It was noted severally that based on the African cultural dynamics and gender roles, men often took the lead, with less transparency and communication with their spouses. A participant explained: “I think that’s the basic problem is communication; that is what African’s lack, that’s where the Caucasians are okay because they talk, Africans don’t talk” (EDF006). The participant elaborated further on how the need to be transparent about finances in Canada changed this dynamic and allowed for more open conversation between spouses:

“… if there is a good communication network between a wife and a husband everything should be put on the table because it is a nuclear family. Like my husband’s family is my family, my family is his family, and of course, if we know the needs, if we are able to be transparent with the needs of all our families. And then we come together with our income, with what we can afford, what we can give and we talk about it, we know how much we have, we know what my family needs, he knows what his family needs and then we have an agreement; there won’t be a problem. But because of lack of trust and because of how African families have always been, the man has always been the provider”.

(EDF006)

In addition, the study revealed the importance of general open communication with regards to personal, family or career tensions that may affect spousal relationships in order to maintain good gender relations within the home. For instance, conversations covered the importance of openness, such as sometimes not being aware of a spouses’ work tensions and challenges which may be affecting their behaviour in the home, which may cause friction unless it is discussed. One participant elaborates:

“but the thing is openness—be open about it. If something is bothering you, talk about it. And I usually tell my husband like if you have a challenge at work or I have a challenge at work, telling you might not help but letting you know it’s good, because sometimes I might be talking to you full of anger, not because you’ve done anything to me but because of what is going on at my workplace. But if you know, you might be able to understand me or deal with the situation much better. But if you don’t know, then you’ll blame me for everything, and you’ll also react in the same way. So I don’t know—we are still working around this area, but it is a very sensitive area, and openness and discussion is really key. Yeah”.

(EDF 005)

Participants also noted that the communication approach was important in maintaining and improving upon communications. They noted the importance of being sensitive to spouses’ trigger points and being respectful in conversation approaches so as not to contribute to the stress and pressures of carrying family responsibilities and the roles each partner played in the home.

“know what your partner likes, even in talking about finances you have to respect your partner’s likes and dislikes, you understand. Like you can have your wife, who really likes to dress up, she likes to spend money, yes, she pays, respects that. And the wife, you who likes to spend money on clothes also know how much your family finances are, you understand, come to an agreement, let there be a balance. As long as we don’t do that, give problem, we have, trust me it’s communication, financial communication, just communicate. It doesn’t mean that I’m going to take all my money and give to you or you’re going to take all your money and we are all together in this thing, it’s a marriage, right …”

(EDF006)

### 3.5. The Need to Set Boundaries

Beyond establishing boundaries in relation to providing financial support, the need to also set boundaries in regard to the influence and interference of the extended family in the nuclear family dynamics was frequently mentioned by participants. Given that the African culture is largely communal, there was a tendency for interference with issues amongst spouses, which more often than not reinforced gender stereotypes. Participants noted that setting boundaries within the context of their relationship and the involvement of extended family members, as well as communicating this clearly to all parties, also improved gender relations. A participant described how establishing boundaries worked in her home:

“I mean if I have issues with my husband, my mother is not the first person I am calling; and this is because for me I do believe strongly in God … and we had an agreement that if we ever have issues, we’re going to talk it through between ourselves first, so before—we rarely bring third parties into our issues, even our extended family, so the boundaries are there. And from my own part and his own part, I think we—both families understand that, that those boundaries are there. We might be quarreling and somebody from our families comes in, you can’t tell we’re quarreling because it’s not for you to know, we’ll deal with that. So that way there’s not been much difference for me when we got to Canada, because from Nigeria, we’ve not really allowed too much influence from extended family”.

(EDF013)

Another participant narrated how family influence had resulted in tensions and the break-up of her friend’s marriage, while noting that she had not had any experience as family relations often differed, but also highlighting the importance of boundaries:

“The experiences are different, different from each family. Most families do not even like that the extended families come. Because my friends have experienced very bad experiences in their own marriages and they’re broken up. So the thing is only that if I had a broken home I will have a good example of that, but now that I don’t have a broken home, that’s why I don’t have a good example of those extended family influences”.

(CAF009)

This shows that while family networks are crucial in the diaspora, it is important that they are managed properly and that communications with all parties are transparent regarding their dynamics, capacity and needs.

## 4. Discussion

In this study, we explored how cultural roots and transnational ties affected/influenced newcomer transitions and integration in Canada and the results highlighted four major intersecting points of influence and confluence, focusing primarily on the female perspectives of SSAIRs. From this perspective, it appears that transnational ties and post-colonial constructs play critical roles in relationships with family and the communal approach to support, as well as in gender relations between SSAIR couples in managing the burden of care and privileges associated with men and communication [16]. Based on the transnational perspective, the importance of maintaining relational ties with the extended family in the homeland has been highlighted as both a source of support—providing moral, social, religious and cultural support during the integration processes—and strain, with participants noting its impact on their integration processes, such as delaying the ability to restructure life, financially plan and save [28]. Although the degree of impact and specificity differs based on slight cultural differences across the SSA region, the commonalities and the range of impact on their lives from managing external family relations were the same for all participants. Our findings are in alignment with these earlier works.

Overwhelmingly, SSAIR females have reported relatively positive changes in the relationships between spouses and with extended family relations upon migration. Notable among these were increased financial independence, improved communications stemming from the need to jointly provide for and manage the family’s affairs as opposed to the culturally imposed presumption of that being a privilege and burden for men, as well as the ability to better manage family interference and interaction due to distance and setting boundaries [29,30]. Although the conclusions are relatively positive, two main challenges were highlighted repeatedly: (1) the strain of shifting gender roles, and (2) the financial burden of maintaining transnational ties with extended family. These two factors contributed to the slowness of the integration process for SSAIRs who were unable to rebuild their lives at a much faster pace.

The study shows that the sense of responsibility towards the extended family that SSAIRs felt was deeply rooted. While many highlighted the importance of setting boundaries, they still considered providing financial support to extended family in their home country an inescapable duty [31]. Additionally, although the study showed shifting gender roles within the family towards more balanced roles, it also revealed a reluctance to embrace this shift. Many participants went on to note that, upon immigration, they have had to play a more prominent role in providing for their families, yet they also spoke about how most of the responsibilities were still the burden of the men [32]. This further illustrates the difficulty in moving past postcolonial ideals of gender relations to transition to more equal gender roles and responsibilities, as well as integration into Canadian society.

The socio-economic pressures experienced by extended families allow immigrant Africans to rethink the distribution of gender roles in the family [11]. This redistribution of roles often requires improved and open communication, resulting in women taking on more financial roles, which thereby provides a sense of responsibility and participation in the decisions regarding the family [33]. The result of this is improved communication between spouses, which contributes to shifting gender roles, as most women, based on their economic contributions and more prominent role in the family finances, have more input in family management, unlike what was the case when in Africa. This is a reinforcing cycle that creates a more balanced gender relation in the family. Furthermore, the increased participation of women in the labour market and as income earners also contributes to the shifting dynamic. Traditionally in most African countries, the women are the homemakers and often run businesses (shops) as their economic activity, which often mean that income may or may not be a steady stream for females. However, as Canadian immigrants, most African women embark on salaried employment, which gives them a steady stream of income that allows them to contribute to and participate in family finances and decisions [34]. Thus, clear communication between spouses helps in this regard and reduces tension that may arise from shifting gender roles. Nonetheless, it appears that although many African immigrant women in Canada contribute more to the family upkeep financially, most final decisions and the heavier burden of finances are still made/shouldered by the men.

The majority of programmes and policies provided to ease integration into Canadian society often focus on economic integration—jobs and financial literacy regarding credits, bills and banking, etc. [35]. Given that a significant challenge to the economic integration of SSAIR women in particular stems from cultural and social ideals from their home countries, perhaps policies and programmes also need to address these issues by upgrading their policies and programs to ease cultural integration and improve gender relations that may arise due to assimilation in Canadian society. Often, when shifting gender roles are not properly managed, this creates further strain on the overall integration processes of SSAIRs, which might create dependence on government support [18]. There need to be enough programmes to prepare immigrants for what to expect culturally, socially and beyond.

Most of our female participants acknowledged the importance of having strong relations with their extended families and feeling responsible for financially supporting them. The findings of our study lead us to recommend that African immigrant families should create open and trusting relationships between partners to avoid conflict involving extended family relations [36]. Moreover, being transparent with family members regarding finances and the challenges of employment in Canada can avoid creating conflict among extended families. Furthermore, there is a need to set up boundaries with extended family members and help them financially within sustainable means and based on the availability of resources to make families self-sufficient, rather than making them dependent [37].

Although this study provided initial information about extended family relations among SSAIRs from the female perspective, future research should focus in greater depth on several of the findings noted in this study. In particular, immigration history (foreign vs. Canadian born, years in Canada) and socioeconomic status (income and education differences) are especially central to issues of immigration and family support and these warrant additional study. Furthermore, the findings of our study underscore the need to strengthen informal support systems for women, such as elders and community leaders. Further investigations can provide insights into the impact of extended family support on family relations and functioning.

## 5. Conclusions

Globalization has fostered new forms of migration as Africans seek better economic opportunities in different parts of the world, predominately in North America. In this study, we examined African immigrants and refugee families’ experiences of extended family support systems from back home through female participants’ perspectives. Our findings lead us to propose the need for interventions focused on strengthening extended family relations in this population. Potential strategies of such interventions include addressing open and trusting relationships among partners, attending to the role of gender, and equipping African immigrant females with skills for dealing with families back home, for example, by setting boundaries, all of which have the potential to improve family harmony and help to avoid conflicts. Support from socially and culturally appropriate programs and support from community partners that attend to these challenges will likely improve the extended family relations of African immigrants living in Canada.

## Data Availability

Not applicable.

## References

[1] Echeverria-Estrada C., Batalova J. (2019). Sub-Saharan African Immigrants in the United States.

[2] Statistics Canada (2019). Diversity of the Black Population in Canada: An Overview.

[3] Statistics Canada (2017). Immigration and Ethnocultural Diversity: Key Results from the 2016 Census.

[4] Madibbo A. (2016). The Way Forward: African Francophone immigrants negotiate their multiple minority identities. J. Int. Migr. Integr..

[5] Comacchio C. (1999). The Infinite Bonds of Family: Domesticity in Canada 1850–1940.

[6] Damaske S., Bratter J.L., Frech A. (2017). Single mother families and employment, race, and poverty in changing economic times. Soc. Sci. Res..

[7] Magalhaes L., Carrasco C., Gastaldo D. (2010). Undocumented migrants in Canada: A scope literature review on health, access to services, and working conditions. J. Immigr. Minority Health.

[8] Bushell R., Shields J. (2018). Immigrant Settlement Agencies in Canada: A Critical Review of the Literature through the Lens of Resilience.

[9] Basch L., Schiller N.G., Blanc C.S. (2020). Nations Unbound: Transnational Projects, Postcolonial Predicaments, and Deterritorialized Nation-States.

[10] Thompson P., Bauer E. (2000). Jamaican transnational families: Points of pain and sources of resilience. Wadabagei J. Caribb. Its Diaspora.

[11] Taylor R.J., Forsythe-Brown I., Lincoln K.D., Chatters L.M. (2017). Extended family support networks of Caribbean Black adults in the United States. J. Fam. Issues.

[12] Gussler J., Barrow C. (1998). Adaptive strategies and social networks of women in St. Kitts. Family in the Caribbean: Themes and Perspective.

[13] Corley A., Sabri B. (2021). Exploring African immigrant women’s pre-and post-migration exposures to stress and violence, sources of resilience, and psychosocial outcomes. Issues Ment. Health Nurs..

[14] Dirlik A. (2018). The Postcolonial Aura: Third World Criticism in the Age of Global Capitalism.

[15] Rothstein-Fisch C., Greenfield P., Quiroz B. (2017). Continuum of “Individualistic” and “Collectivistic” Values.

[16] Guyo F.B. (2017). Colonial and post-colonial changes and impact on pastoral women’s roles and status. Pastoralism.

[17] Bahri D. (2009). Feminism and Postcolonialism in a global and local frame. Vents d’Est, Vents d’Quest: Mouvements de Femmes et Feminismesanticoloniaux.

[18] Breines I., Connell R., Connell R., Eide I. (2000). Male Roles, Masculinities and Violence: A Culture of Peace Perspective.

[19] Walton-Roberts M. (2004). Transnational migration theory in population geography: Gendered practices in networks linking Canada and India. Popul. Space Place.

[20] Mazzucato V., DeWind J., Holdaway J. (2008). Simultaneity and Networks in Transnational Migration: Lessons Learned from an SMS Methodology.

[21] Sen G., George A., Ostlin P., Ramos S. (2007). Unequal, Unfair, Ineffective and Inefficient Gender Inequity in Health: Why It Exists and How We Can Change It.

[22] Boyd M., Grieco E. (2014). Women and Migration: Incorporating Gender into International Migration Theory.

[23] Kesby M. (2005). Retheorizing empowerment-through-participation as a performance in space: Beyond tyranny to transformation. Signs J. Women Cult. Soc..

[24] Cornwall A., Jewkes R. (1995). What is participatory research?. Soc. Sci. Med..

[25] Minkler M., Wallerstein N. (2003). Part one: Introduction to community-based participatory research. Community-Based Participatory Research for Health.

[26] Braun V., Clarke V. (2006). Using thematic analysis in psychology. Qual. Res. Psychol..

[27] Guba E.G., Lincoln Y.S. (1994). Competing paradigms in qualitative research. Handb. Qual. Res..

[28] Baldassar L. (2007). Transnational families and the provision of moral and emotional support: The relationship between truth and distance. Identities Glob. Stud. Cult. Power.

[29] Mahler S.J. (2001). Transnational relationships: The struggle to communicate across borders. Identities Glob. Stud. Cult. Power.

[30] Hughes N., Munoz-Guzman C. (2016). Understanding and Supporting ‘Families with Complex Needs’. Social Sciences, Printed edition of the Special Issue Understanding and Supporting ‘Families with Complex Needs’. http://www.mdpi.com/journal/socsci/special_issues/families.

[31] Thomas D. (2001). Evolving family living arrangements of Canada’s immigrants. Can. Soc. Trends.

[32] Flahaux M.L., De Haas H. (2016). African migration: Trends, patterns, drivers. Comp. Migr. Stud..

[33] Cerrato J., Cifre E. (2018). Gender inequality in household chores and work-family conflict. Front. Psychol..

[34] Do D. (2020). Canada’s Black Population: Education, Labour and Resilience. Ethnicity, Language and Immigration Thematic Series.

[35] George G., Selimos E.D. (2018). Using narrative research to explore the welcoming of newcomer immigrants: A methodological reflection on a community-based research project. Forum: Qualitative Social Research.

[36] Okeke-Ihejirika P. (2020). Integration of African Immigrants to Alberta (Challenges and Recommendations).

[37] Okeke-Ihejirika P., Salami B., Karimi A. (2019). African immigrant women’s transition and integration into Canadian society: Expectations, stressors, and tensions. Gend. Place Cult..

